# Autochthonous *Leishmania infantum* in Dogs, Zambia, 2021

**DOI:** 10.3201/eid2804.212378

**Published:** 2022-04

**Authors:** David Squarre, Herman M. Chambaro, Kyoko Hayashida, Lavel C. Moonga, Yongjin Qiu, Yasuyuki Goto, Elizabeth Oparaocha, Chisoni Mumba, Walter Muleya, Patricia Bwalya, Joseph Chizimu, Mwelwa Chembensofu, Edgar Simulundu, Wizaso Mwasinga, Nelly Banda, Racheal Mwenda, Junya Yamagishi, King S. Nalubamba, Fredrick Banda, Musso Munyeme, Hirofumi Sawa, Paul Fandamu

**Affiliations:** University of Edinburgh, Edinburgh, Scotland, UK (D. Squarre);; Ministry of Fisheries and Livestock, Lusaka, Zambia (D. Squarre, H.M. Chambaro, P. Bwalya, F. Banda, P. Fandamu);; Hokkaido University, Sapporo, Japan (H.M. Chambaro, K. Hayashida, L.C. Moonga, Y. Qiu, J. Chizimu, J. Yamagishi, H. Sawa);; Central Veterinary Research Institute, Lusaka (H.M. Chambaro, F. Banda);; University of Tokyo, Tokyo, Japan (Y. Goto);; Showgrounds Veterinary Clinic, Lusaka (E. Oparaocha);; University of Zambia, Lusaka (C. Mumba, W. Muleya, M. Chembensofu, E. Simulundu, W. Mwasinga, N. Banda, R. Mwenda, K.S. Nalubamba, M. Munyeme);; Macha Research Trust, Macha, Zambia (E. Simulundu); Global Virus Network, Baltimore, Maryland, USA (H. Sawa)

**Keywords:** leishmaniasis, canine leishmaniasis, Leishmania infantum, parasites, protozoa, autochthonous, dogs, kala-azar, zoonoses, public health, vector-borne infections, sand flies, Zambia

## Abstract

Leishmaniases are neglected tropical diseases of humans and animals. We detected *Leishmania infantum* in 3 mixed-breed dogs in Zambia that had no travel history outside the country. Our findings suggest presence of and probable emergence of leishmaniasis in Zambia, indicating the need for physicians and veterinarians to consider the disease during diagnosis.

Leishmaniasis, a neglected tropical disease of humans and animals, is estimated to affect <1 million persons annually (https://www.who.int/health-topics/leishmania). The disease is caused by intracellular protozoan parasites of the genus *Leishmania* (Trypanosomatida: *Trypanosomatidae*), which are vectored by female sand flies of the genera *Phlebotomus* in the Old World and *Lutzomyia* in the New World (*1*). Although leishmaniasis is endemic to several resource-poor countries in eastern, western, and northern Africa, there is a dearth of information on the epidemiology of the disease in southern Africa, largely caused by weak surveillance systems (*1**,**2*).

In Zambia, human visceral leishmaniasis was reported in 1973 in the Eastern Province (*3*) and subsequently in 1976 in the same area (*4*). In 1994, a case of canine visceral leishmaniasis was reported in a dog in Lusaka Province (*5*). We report detection of canine leishmaniasis caused by *Leishmania infantum*, suggesting possible reemergence or reintroduction of the disease in Zambia. 

The study was approved by the Department of Veterinary Services, Government of the Republic of Zambia. In June 2021, two female mixed-breed shelter dogs (case 1 and case 2) rescued in Southern Province of Zambia during 2020 were brought to a veterinary clinic in Lusaka. These 2 dogs had chronic weight loss, generalized alopecia, and ulcerative and exfoliative dermatitis (Figure, panel A). The dogs had been previously treated for tickborne and helminth infections but showed no improvement. Physical examination showed that the prescapular and popliteal lymph nodes were enlarged. Case 1 had onychogryphosis (Figure, panel A) and focal corneal opacity in the left eye (Figure, panel B). 

**Figure:**
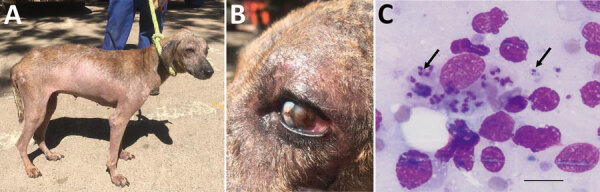
Clinical manifestations and microscopic imaging of *Leishmania infantum*‒infected dog, Zambia. A, B) Dog (case 1) showing dermatitis and onychogryphosis (excessive growth of nails) (A) and focal corneal opacity of the left eye (B). C) Intracellular *Leishmania* amastigotes (black arrows) in fine-needle lymph node aspirate from the same dog. Scale bar indicates 20 μm.

Biochemistry profiles for both dogs showed increased levels of serum proteins (>93.3 g/L) and hyperglobulinemia (>74.9 g/L) and hypoalbuminemia (<14.7 g/L), which are common manifestations suggestive of canine leishmaniasis. Giemsa staining of fine-needle lymph node aspirate identified *Leishmania* spp. amastigotes (Figure, panel C).

We performed serologic analysis of *Leishmania* antibodies by using *L. donovani* soluble lysate antigen from cultured promastigotes and recombinant LinJ14.1160r4 antigens as described (*6*). Both dogs had high antibody titers, >1.0 optical density units (Appendix Figure). Among in-contact dogs from Southern Province that had no clinical signs (n = 6), 1 dog (case 3) had high antibody titers for both assays, and 2 dogs (dogs 6 and 7) had high antibody titers for LinJ14.1160r4 only. All control serum samples (n = 39) from Central Province were negative for *Leishmania* antibodies on both assays (Appendix Figure). For the purpose of disease control and the absence of antileishmanial agents in Zambia, the dogs (cases 1 and 2) were euthanized by rapid intravenous infusion of pentobarbitone sodium (0.7 mL/kg body weight). Case 3, an in-contact dog that showed high antibody titers for both assays, was euthanized 1 month after clinical disease was detected and the initial diagnosis.

At necropsy, we aseptically harvested spleen tissue and processed this tissue for genomic DNA extraction by using the QuickGene DNA Tissue Kit (Kurabo, https://www.kurabo.co.jp) according to the manufacturer’s protocol. We performed PCRs targeting the partial small subunit ribosomal RNA gene (*7*) and internal transcribed spacer (ITS) 1 and ITS 2 genes (*8*). PCR for 3 dogs showed expected band sizes, which we purified and sequenced on a 3500 Genetic Analyzer (Applied Biosystems, https://www.thermofisher.com). Sequences obtained were 100% identical with the *L. infantum* reference strain (JPCM5) isolated in Spain (*9*). The ITS sequence type was type A, which was assigned according to 12 microsatellite regions in ITS1 and ITS2 within the *L. donovani* complex (*8*). ITS type A is the dominant *L. infantum* type reported mainly from the Mediterranean basin, and types D, E, F, and G are associated with *L. donovani* from eastern Africa (*8*). Nucleotide sequences from this study were deposited in the DNA DataBank of Japan (GenBank accession nos. LC652643‒LC652645).

Our study confirmed the presence and probable emergence of leishmaniasis and *Leishmania* parasites in Zambia. An in-contact, seropositive dog that did not have clinical signs had clinical disease develop 1 month after the initial diagnosis. However, the probable route of infection remains unclarified.

Although the geographic distribution of vector sandflies has not been described in Zambia, neighboring countries have reported presence of *Phlebotomus* spp. sand flies (*10*). In addition, although the extent of disease distribution in the country, including Southern Province, is yet to be determined, autochthonous leishmania cases reported in Zambia (*3**–**5*) suggests the presence of an infection foci. To further clarify the epidemiology of leishmaniasis in Zambia, there is need for improved understanding of the epidemiology of the disease in dogs, vector distribution, and the risk for human infection, particularly in high-risk populations, such as immunocompromised persons.

Appendix Additional information on autochthonous *Leishmania infantum* in dogs, Zambia, 2021.
