# Supplementary material for: Autochthonous Leishmania infantum in Dogs, Zambia, 2021
Source: Emerg Infect Dis. 2022 Apr;28(4):888–90. doi: 10.3201/eid2804.212378 (PMC8962896; doi:10.3201/eid2804.212378)
Supplement: Appendix — Additional information on autochthonous Leishmania infantum in dogs, Zambia, 2021. [file 21-2378-Techapp-s1.pdf]

Article DOI: https://doi.org/10.3201/eid2804.212378

Autochthonous *Leishmania infantum* in Dogs, Zambia, 2021

Appendix

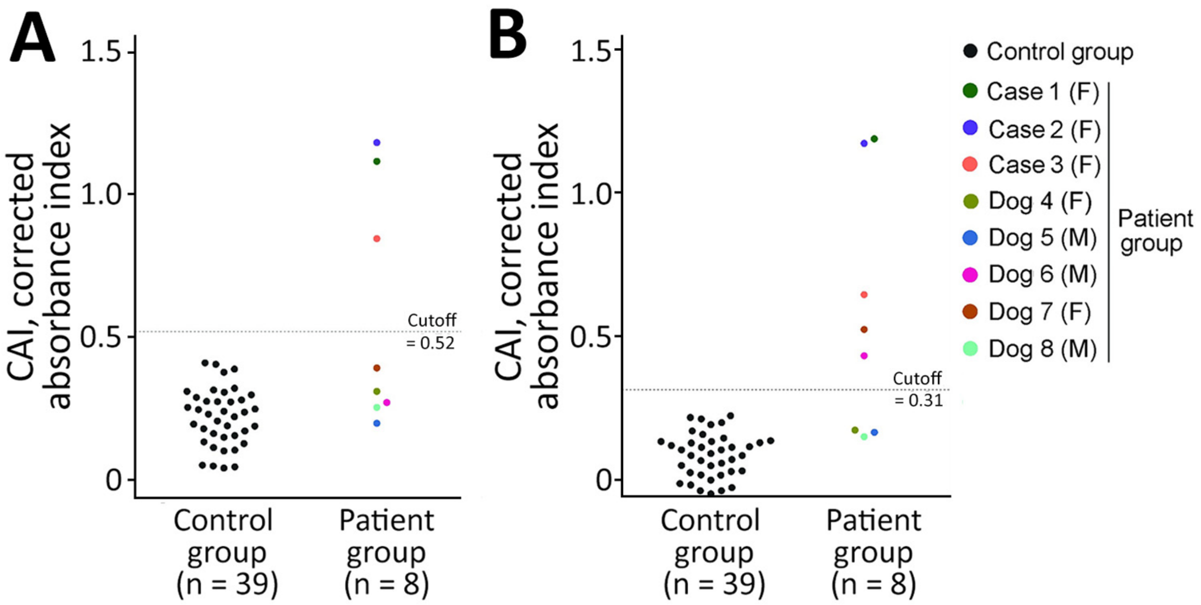

Reference

1. Goto Y, Carter D, Guderian J, Inoue N, Kawazu S, Reed SG. Upregulated expression of B-cell antigen family tandem repeat proteins by *Leishmania* amastigotes. Infect Immun. 2010;78:2138–45. PubMed https://doi.org/10.1128/IAI.01102-09
